# Low radial and axial force stent retriever reduces symptomatic subarachnoid hemorrhage after mechanical thrombectomy for acute middle cerebral artery and medium vessel occlusion: a prospective pilot study

**DOI:** 10.3389/fneur.2025.1723287

**Published:** 2026-01-12

**Authors:** Taichi Ishiguro, Yoshihiro Omura, Yuki Takano, Takashi Arai, Kostadin Karagiozov, Kotaro Fukuda, Yoshichika Kikuta, Nobuhiko Momozaki, Hiroki Eguchi, Masahiko Nishitani, Makiko Sakaguchi, Momo Uchida, Mana Suzuki, Takakazu Kawamata

**Affiliations:** 1 Department of Neurosurgery, Tokyo Women's Medical University Yachiyo Medical Center, Chiba, Japan; 2 Department of Radiology, Tokyo Women's Medical University Yachiyo Medical Center, Chiba, Japan; 3 Department of Neurosurgery, Tokyo Women’s Medical University Hospital, Tokyo, Japan

**Keywords:** axial force, mechanical thrombectomy, medium vessel occlusion, middle cerebral artery occlusion, radial force, stent retriever, subarachnoid hemorrhage

## Abstract

**Background:**

Subarachnoid hemorrhage (SAH) is a well-recognized complication after mechanical thrombectomy (MT) and may adversely affect clinical outcomes. SAH commonly results from vessel injury due to overextension or displacement during device retrieval. This risk is particularly concerning in smaller, tortuous vessels, such as in medium vessel occlusions (MeVO). This study evaluated whether a novel low radial and axial force stent retriever (Tron FX II) could reduce post-procedural SAH without compromising recanalization outcomes.

**Methods:**

The study comprised two components: (1) bench testing comparing radial/axial force and vessel displacement during retrieval between Tron FX II (hereafter Tron) and conventional devices; and (2) a prospective observational study of 197 consecutive MT patients. A combined technique was employed in all cases. Conventional stent retrievers were used until July 2023, after which Tron was used exclusively. Post-procedural SAH was assessed using dual-energy CT immediately after MT. The primary outcome was SAH incidence, with or without symptoms. Secondary outcomes included effective recanalization, first-pass rate, and 90-day functional outcomes.

**Results:**

Bench testing showed Tron exhibited the lowest radial/axial force and the smallest vessel deviation during retrieval. Clinically, Tron significantly reduced the incidence of post-procedural SAH (7.7% vs. 23.9%, *p* = 0.027) as well as SAH associated with neurological deterioration (1.9% vs. 12.7%, *p* = 0.044) in cases of middle cerebral artery occlusion (MCAO) and MeVO. Moreover, both Tron use and fewer stent retriever passes were independently associated with a lower risk of SAH. Overall effective recanalization, first-pass rates, and functional outcomes were not significantly different between groups in this pilot study.

**Conclusion:**

The low radial and axial force stent retriever reduced post-procedural SAH while maintaining effective recanalization when combined with an aspiration catheter. These findings support its potential role as a safer option for MCAO and MeVO.

## 1 Introduction

In recent years, mechanical thrombectomy (MT) has become an essential treatment for acute ischemic stroke, particularly in patients with large vessel occlusions (1–3). However, intracranial hemorrhage, particularly subarachnoid hemorrhage (SAH) remains a significant complication following MT and may adversely affect clinical outcomes (4–6). This issue is particularly important in cases of medium vessel occlusion (MeVO). Recent studies, including the ESCAPE-MeVO and DISTAL trials, have shown no functional advantage of MT over best medical therapy, with the excess intracranial hemorrhage in the MT groups largely accounting for worse outcomes (7, 8). Consequently, identifying optimal devices and techniques to minimize hemorrhagic complications after MT is a critical and ongoing objective.

Among the various MT strategies, stent retrievers are widely used due to their ability to achieve rapid and effective recanalization. One major cause of post-procedural SAH is believed to be vessel injury, particularly vessel perforation, caused by vessel wall overextension or displacement during retrieval (4, 5, 9). This risk is often attributed to the strong radial force inherent in the design of current stent retrievers, which is intended to ensure robust thrombus engagement (10). However, in combined techniques, in which a stent retriever is used in conjunction with an aspiration catheter, the primary role of the stent retriever is changed. In such cases, the device primarily serves to anchor and stabilize the aspiration catheter, support thrombus capture, and prevent distal embolization (11, 12). In this context, high radial force may be unnecessary and potentially harmful.

To address these concerns, we employed a novel stent retriever with reduced radial and axial force, specifically designed to minimize vessel wall deformation during retrieval and thereby reduce the risk of vascular injury and SAH. Simultaneously, the device maintains sufficient structural integrity to support aspiration catheter navigation and facilitate effective thrombus capture. As supporting evidence for our hypothesis has not previously been reported in the literature, we conducted bench testing of the mechanical parameters of the novel stent retriever. Subsequently, the performance of the novel stent retriever was clinically evaluated in a prospective cohort study, and the incidence of hemorrhagic complications and clinical outcomes were compared to those associated with existing commercial devices.

## 2 Materials and methods

### 2.1 Stent structure

**Figure 1:**
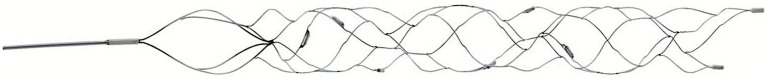
Structure of the low radial/axial force stent retriever (Tron FX II) showing the hybrid cell design to enhance flexibility and reduce vessel wall injury.

The novel low radial and axial force stent retriever (Tron FX II, Otsuka Medical Devices Co., Ltd., Tokyo, Japan) was designed to enhance flexibility and reduce mechanical stress by limiting the stent’s contact area with the vascular endothelium (Figure 1). The device features a hybrid cell design composed of two distinct cell structures. It is engineered to capture thrombi by engaging them within the stent lumen during navigation through the cerebral vasculature (13).

### 2.2 Bench testing protocol

We conducted bench testing of the mechanical parameters of Tron FX II 4 × 40 mm and compared the results with three widely used stent retrievers; Solitaire™ X Revascularization Device 4 × 40 mm (Medtronic, Minneapolis, MN, USA), Trevo™ NXT ProVue Retriever 4 × 28 mm (Stryker, Kalamazoo, MI, USA), and EmboTrap™ III Revascularization Device 5 × 37 mm (CERENOVUS, Irvine, CA, USA).

#### 2.2.1 Radial force measurement

**Figure 2:**
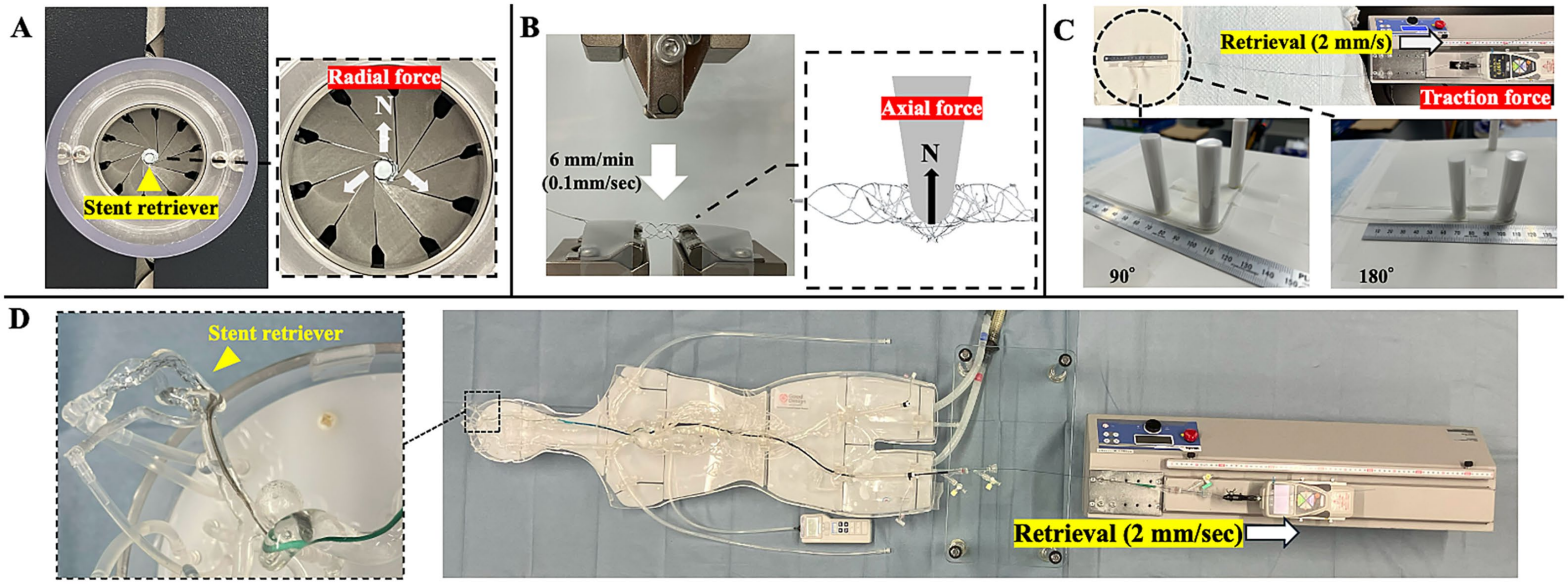
Bench testing protocols for evaluating the mechanical characteristics of stent retrievers. **(A)** Radial force measurement using the TTR2 radial force testing system. The stent retriever was placed in the test chamber, radially contracted to 0.5 mm, and then expanded at 0.5 mm/s. Outward radial force was continuously recorded during expansion. **(B)** Axial force measurement setup. The stent was fixed on a jig for three-point bending. A flat pusher was lowered at 0.1 mm/s using a universal testing machine, and the vertical load was recorded during compression. **(C)** Retrieval traction test simulating vessel curvature using silicone tubes with 0°, 90°, and 180° bends (radius of curvature 6 mm), with force recorded during constant-speed retrieval at 2 mm/s. **(D)** Assessment of vessel deviation during stent retriever retrieval. Using a silicone vascular model with pulsatile flow, a stent retriever was deployed in the M2 distal segment and retrieved at 2 mm/s. The maximum vessel deviation was visualized by superimposing images from the initial position and peak displacement.

Radial force is the component of force acting perpendicular to the axis of rotation. Radial force was measured using the TTR2 radial force testing system (Blockwise Engineering LLC, Tempe, AZ, USA). Each stent retriever was placed in a test chamber maintained at 37 ± 2 °C. The chamber was first radially contracted until the diameter reached 0.5 mm to simulate the condition immediately after deployment from a microcatheter. The diameter was then gradually increased at a rate of 0.5 mm/s, which was selected as the most stable and reproducible speed based on preliminary testing. The outward radial force was continuously recorded during the expansion of the device (Figure 2A).

#### 2.2.2 Axial force measurement

Axial force, defined as the force exerted by a stent along the vessel’s length to straighten a curved configuration, was measured using a universal testing machine (Autograph AGS-X, Shimadzu Corporation, Kyoto, Japan). Each stent retriever was preheated in a water bath maintained at 37 °C and then fixed onto a jig for three-point bending using adhesive tape. A flat pusher was positioned vertically on top of the stent and lowered at a constant speed of 0.1 mm/s (Figure 2B). This speed was selected in accordance with ASTM F2606, which recommends displacement-controlled testing for vascular stents and is supported by prior studies showing minimal speed dependence of axial resistance within the 1–10 mm/min range (14). The vertical load applied to the stent was recorded during compression.

#### 2.2.3 Retrieval traction test

The retrieval traction test was conducted to simulate vessel curvature and quantitatively evaluate the retrieval force required for stent retrievers under varying anatomical conditions. The test was performed using a tensile testing machine (MH2-500 N-EX-05246, IMADA CO., LTD) equipped with a force gauge (ZTA-20 N, IMADA CO., LTD.). Each device was preheated in a 37 °C water bath and then deployed into a silicone tube with an inner diameter of 1.5 mm. The wire of the device was connected to the arm of the force gauge. The tube was filled with warm water maintained at 37 °C. The device was retrieved at a constant velocity of 2 mm/s, and the maximum traction force generated during retrieval was recorded in newtons. The test was repeated three times under the following bending configurations of the silicone tube—0° (straight), 90° (right angle), and 180° (U-shaped bend)—with a fixed radius of curvature of 6 mm (Figure 2C).

#### 2.2.4 Vessel shift evaluation induced by stent retrieval

To visualize and assess how each stent retriever influences vessel deviation during mechanical thrombectomy, we conducted experiments using a catheter simulation system equipped with a silicone vascular model (EndoVascular Evaluator; EVE, FAIN-Biomedical, Nagoya, Japan) (15). The EVE system consists of transparent silicone tubing replicating major arteries, including the heart, and is designed as a closed-loop circuit. Its CT-based vascular geometry (~100-μm accuracy) and silicone vessel properties (Young’s modulus 1.87–1.9 MPa, Poisson’s ratio 0.46, friction coefficient 0.042) closely match human arterial tissue, enabling realistic simulation of M2 vessel deformation and friction (16–18). A motor-connected pump generates pulsatile flow by ejecting fluid from the heart chamber, thereby dynamically reproducing systemic blood circulation. The artificial vessel circuit was filled with 10 liters of tap water containing BIOACT (FAIN-Biomedical), a surfactant solution used to enhance catheter lubricity. The experimental system was operated at a pressure of 120 mmHg and a flow rate of 7.5 L/min, parameters that closely approximate physiological hemodynamics.

To further simulate the intraoperative scenario, a 9 Fr. Optimo balloon-guiding catheter (Tokai Medical Products, Aichi, Japan) was placed in the internal carotid artery during testing to maintain systemic arterial flow while selectively reducing cerebral arterial flow. A stent retriever was deployed in a way that its distal tip was placed in the M2 distal segment via a Headway 21 microcatheter (Terumo Neuro, Aliso Viejo, CA, USA) and subsequently retrieved at a constant speed of 2 mm/s using a tensile testing machine (MH2-500 N-EX-05246, IMADA CO., LTD., Toyohashi, Japan). The retrieval procedure was video-recorded, and still images were generated by superimposing the baseline vessel silhouette with the frame showing maximum vessel displacement (Figure 2D). Vessel deviation was quantified by measuring the displacement of five predefined vascular landmarks to determine the extent of vessel movement (19).

### 2.3 Clinical evaluation

#### 2.3.1 Study design

This prospective observational study included 232 consecutive patients who underwent MT for acute ischemic stroke between April 2021 and March 2025 at our institution. Beginning in July 2023, the Tron FX II stent retriever (hereafter referred to as Tron) was exclusively used for all eligible cases (Tron group). Outcomes in this group were compared with those of patients treated before July 2023 (control group). Patients who underwent aspiration-only thrombectomy (i.e., without the use of a stent retriever) and those with intracranial atherosclerotic disease who required percutaneous transluminal angioplasty or stenting during MT were excluded from the analysis.

We collected data on baseline clinical characteristics, including age, sex, comorbidities (hypertension, diabetes mellitus, coronary artery disease, and smoking status), and neurological severity assessed by the National Institutes of Health Stroke Scale (NIHSS) on admission (20).

Radiological characteristics included infarct burden on non-contrast CT based on the Alberta Stroke Program Early CT Score (ASPECTS) and the site of vessel occlusion (21). In case of posterior circulation occlusion, PC-ASPECTS was evaluated (22). The middle cerebral artery (MCA) was further sub-classified into M1, M2, and M3 or beyond segments. Occlusions located in the MCA M2 segment or beyond, ACA, and PCA were defined as medium vessel occlusion (MeVO), while occlusions involving the internal carotid artery terminus, MCA M1 segment, vertebral artery, or basilar artery were considered large vessel occlusion (LVO). Procedural characteristics included the type of stent retriever used, number of device passes, final angiographic results assessed by the modified Thrombolysis in Cerebral Infarction (mTICI) grading system, and puncture-to-recanalization time (23, 24).

The primary outcome was the incidence of SAH following MT, with or without symptoms. The radiological assessment of SAH is detailed in the following section. Secondary outcomes included the effective recanalization (mTICI ≥2b), first-pass rate, and modified Rankin Scale (mRS) score at 90 days. All variables were compared between the Tron group and the control group using conventional stent retrievers to evaluate differences in hemorrhagic complication rates and clinical outcomes.

#### 2.3.2 Evaluation of subarachnoid hemorrhage

Subarachnoid hemorrhage following MT, the primary outcome of this study, were assessed using follow-up imaging performed just after the procedure. Diagnosis was based on virtual non-contrast images obtained with dual-energy CT, which minimizes the effects of contrast leakage (25, 26). The presence or absence of SAH was assessed in an independent, blinded fashion by three neuroradiologists. Symptomatic SAH was defined as any SAH associated with neurological deterioration, indicated by an increase of ≥4 points on the NIHSS.

#### 2.3.3 Mechanical thrombectomy procedure

We employed the combined technique as the first-line approach for both anterior and posterior circulation. This involved deploying a stent retriever across the thrombus while simultaneously applying continuous aspiration through an aspiration catheter positioned proximally. Once the clot is engaged, the stent retriever and aspiration catheter are withdrawn together, with flow arrest achieved using a balloon guiding catheter to minimize distal embolization. Stent retrievers including Solitaire X, Trevo NXT, and EmboTrap III were used prior to July 2023, while Tron was used exclusively thereafter. Device selection was based on the diameter of the target vessel. If the thrombus was not successfully removed on the first pass, the procedure was repeated using the same devices up to 3 passes.

### 2.4 Statistical analysis

Categorical variables were compared using the chi-square test or Fisher’s exact test as appropriate. Continuous variables with normal distribution were expressed as mean ± standard deviation and analyzed using the t-test. Variables with non-normal distribution were reported as median [interquartile range] and compared using the Wilcoxon rank-sum test. A *p*-value < 0.05 was considered statistically significant. To evaluate the risk of post-procedural SAH associated with the stent retriever type, logistic regression analysis was performed to calculate adjusted odds ratios (ORs) with 95% confidence intervals (CIs). The multivariate logistic regression model included potential confounders: age, sex, baseline NIHSS, ASPECTS, occluded vessel, number of stent-retriever passes, and puncture-to-recanalization time. All analyses were conducted using JMP Pro version 17 (SAS Institute, Cary, NC, USA).

## 3 Results

### 3.1 Bench test results of stent retriever

To clarify the mechanical properties and vessel interactions of various stent retrievers in the context of M2 occlusions, we conducted a series of bench tests comparing Tron FX II (Tron 4–40) with Solitaire™ X Revascularization Device 4 × 40 mm (Solitaire 4–40), Trevo™ NXT ProVue Retriever 4 × 28 mm (Trevo 4–28), and EmboTrap™ III 5 × 37 mm (EmboTrap 5–37).

**Figure 3:**
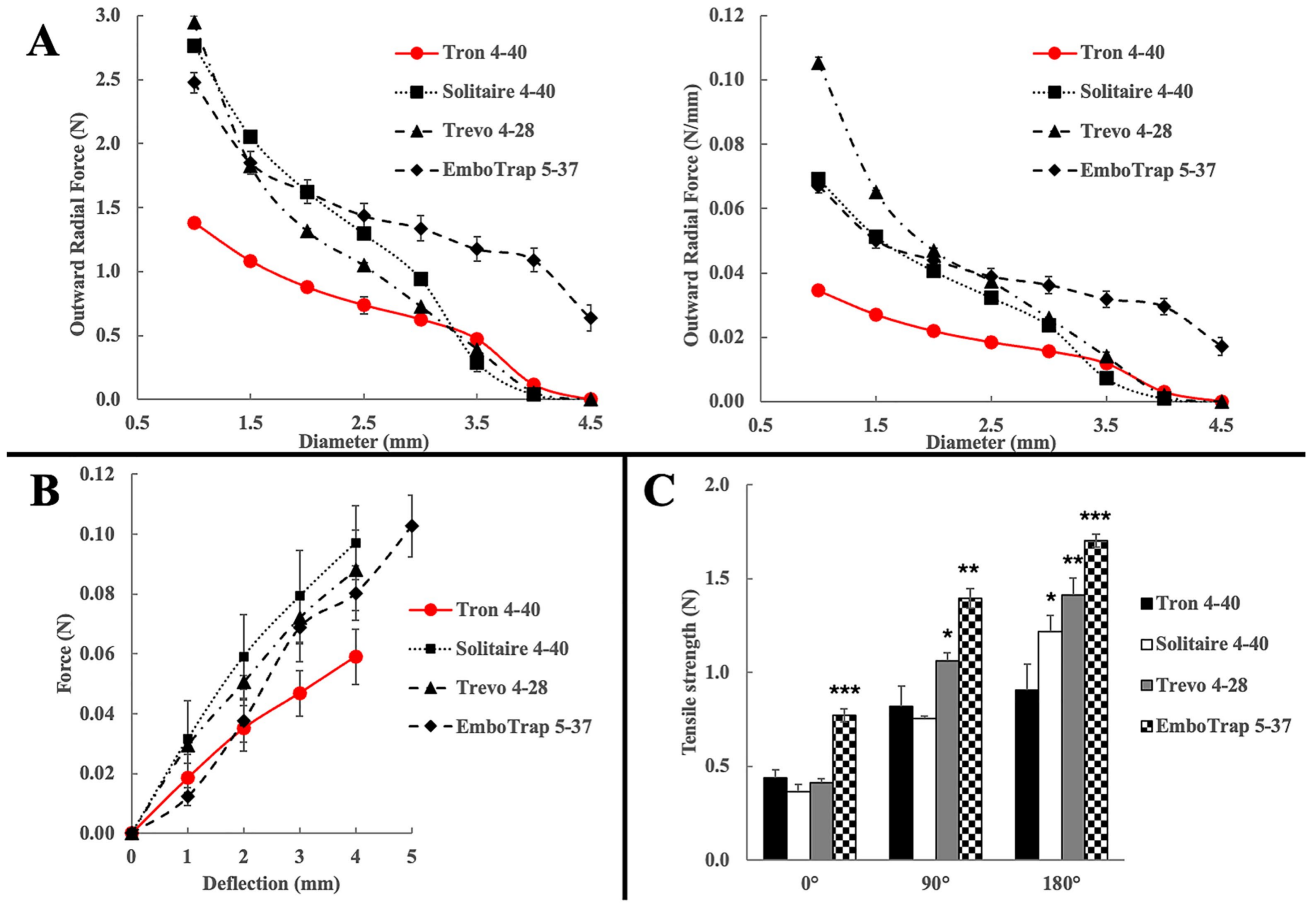
Bench test results comparing Tron FX II with conventional stent retrievers. **(A)** Radial force across deployment diameters (N, right) and radial force per unit length (N/mm, left). Tron shows consistently lower radial force in both assessments, particularly in the 1.0–2.0 mm range corresponding to M2 or medium-caliber vessels. **(B)** Axial flexibility (three-point bending test) demonstrating greater longitudinal flexibility for Tron compared with other devices. **(C)** Retrieval traction force in straight and curved vessel models. Tron demonstrated the lowest traction forces, particularly in 180° curved configurations. *p < 0.05, **p < 0.01, ***p < 0.001 vs. Tron at each corresponding angle.

Radial force was measured to evaluate the extent of radial stress each device applies to small-diameter cerebral vessels. Tron 4–40 consistently exhibited the lowest radial force among all devices (Figure 3A, left), and radial force normalized by stent length (27) similarly remained lowest for Tron 4–40 (Figure 3A, right). Notably, within the vessel diameter range of 1.0–2.0 mm, corresponding to the M2 segment, Tron 4–40 alone demonstrated a significantly lower radial force compared to the other devices.

Axial flexibility, simulating longitudinal deformation during navigation and retrieval, was assessed using a three-point bending test. Tron 4–40 exhibited the highest longitudinal flexibility, indicating superior conformability to tortuous vessels and being less prone to transmitting mechanical stress to the vessel wall (Figure 3B).

Following these radial and axial force assessments, retrieval traction force was further evaluated using a tensile testing system. The devices were pulled through three types of silicone tubes with a fixed curvature radius of 6 mm: 0° (straight), 90° (right-angle), and 180° (U-shape). Under the straight condition, EmboTrap 5–37 showed the highest traction force. In the 90° condition, EmboTrap 5–37 and Trevo 4–28 exerted greater forces than Tron 4–40 and Solitaire 4–40. Under the 180° condition, Tron 4–40 consistently showed the lowest traction force, whereas all other devices exerted significantly greater forces (Figure 3C).

**Figure 4:**
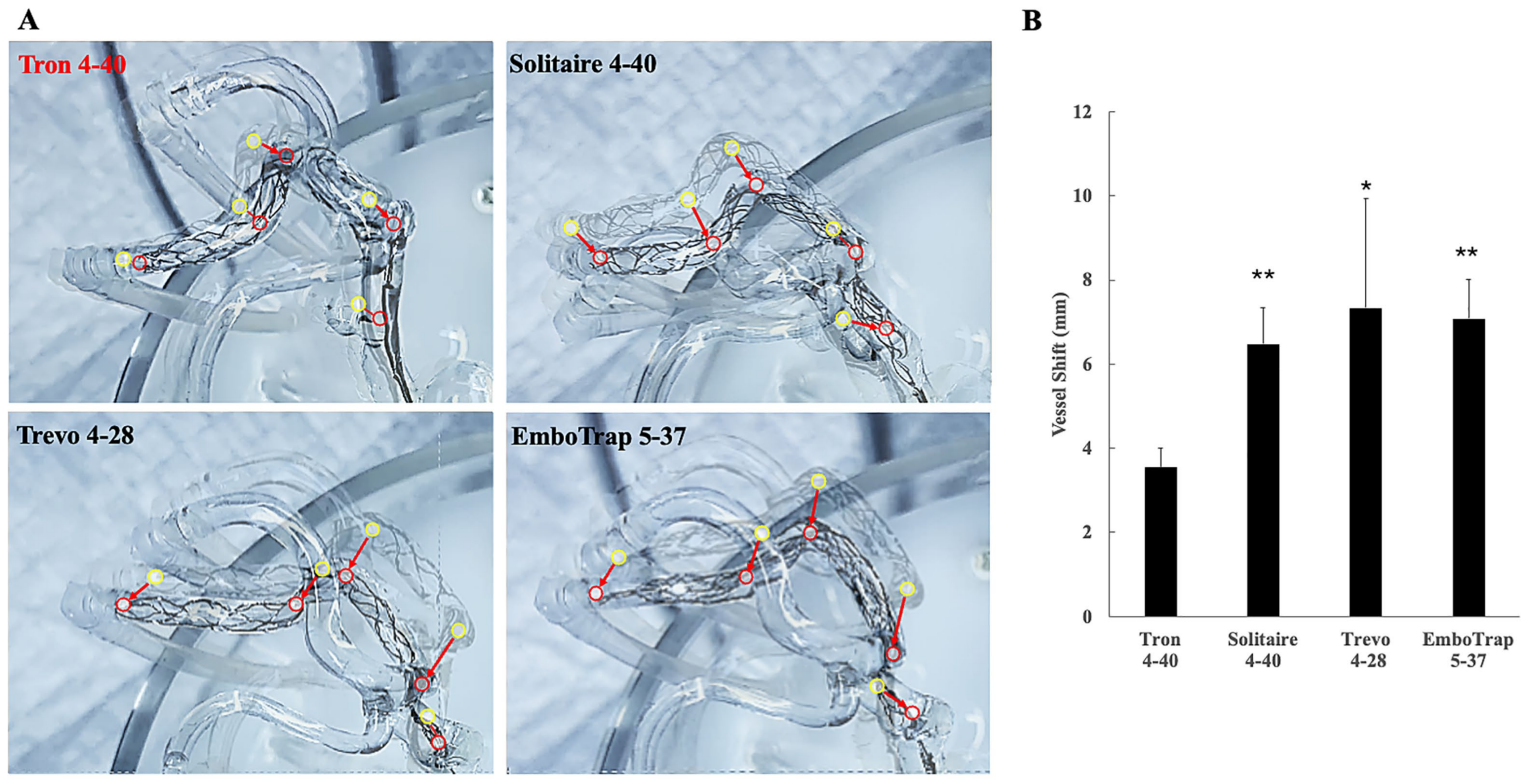
Impact of each stent retriever on vessel deviation during retrieval, assessed using a silicone vascular model (EVE system). **(A)** Representative still images showing the baseline vessel silhouette overlaid with the frame of maximum deviation. Positional changes at five predefined vascular landmarks are illustrated before (yellow circles) and after (red circles) retrieval (arrows). **(B)** Quantitative measurements of vessel deviation at the same five landmarks. Among all devices tested, Tron 4–40 exhibited a significantly smaller degree of vessel shift compared with the other stent retrievers. Bar graphs represent mean displacement (mm) ± SD. *p < 0.05 vs. Tron 4–40; **p < 0.001 vs. Tron 4–40.

Finally, the influence of each stent retriever on vessel shift during retrieval was examined using a silicone vascular model (EVE system). Figure 4A presents still images obtained by superimposing the baseline vessel silhouette with the frame of maximum vessel deviation during retrieval, providing a visual representation of vessel shift. Quantitative measurements of vessel deviation at five predefined vascular landmarks are shown in Figure 4B. Among all devices tested, Tron 4–40 demonstrated a significantly smaller degree of vessel shift than the other stent retrievers.

### 3.2 Clinical outcomes

A total of 197 patients were included in the analysis, comprising 121 patients treated with conventional stent retrievers and 76 patients treated with Tron FX II. Table 1 summarizes the comparison of baseline characteristics and treatment outcomes between the Tron and conventional stent retriever groups. There were no significant differences in preoperative factors, including patient age, sex, pretreatment NIHSS score, ASPECTS, or the distribution of occluded vessels between the two groups. Regarding procedural factors, the incidence of overall SAH (including both asymptomatic and symptomatic cases) was significantly lower in the Tron group (6.6% vs. 19.8%, *p* = 0.012). Notably, 10 of 29 SAH cases (34.5%) were associated with neurological deterioration (defined as an increase of ≥4 points on the NIHSS). The incidence of such clinically significant SAH also tended to be lower in the Tron group (1.3% vs. 7.4%, *p* = 0.092). The neurological deterioration observed in symptomatic SAH was predominantly attributable to concomitant acute hydrocephalus. No significant differences were observed between the two groups in terms of stent retriever size, total number of stent retriever passes, rate of first-pass recanalization, effective recanalization rate, or the proportion of patients achieving a favorable functional outcome (mRS 0–2) at 3 months. However, the puncture-to-recanalization time tended to be shorter in the Tron group.

**Table 1:** Comparison of baseline characteristics and treatment outcomes between the Tron and conventional stent retriever groups.

Variables	Tron (*n* = 76)	Other SRs (*n* = 121)	*p*-value
Age (year), mean (SD)	80.2 (10.5)	79.3 (10.7)	0.716
Female sex (%)	41 (54.0)	56 (46.3)	0.309
NIHSS, mean (SD)	22.7 (8.4)	21.4 (8.9)	0.311
ASPECTS, median (IQR)	9 (7–10)	9 (7–10)	0.117
Occluded vessel (%)			
*ICA*	16 (21.1)	37 (30.6)	0.307
*M1*	36 (47.4)	40 (33.1)	
*M2*	15 (19.7)	27 (22.3)	
*VABA*	8 (10.5)	13 (10.7)	
*ACAPCA*	1 (1.3)	4 (3.3)	
Used stent size (mm), mean (SD)	4.72 (1.07)	4.5 (1.10)	0.156
Number of passes, mean (SD)	1.8 (0.95)	1.88 (1.17)	0.634
First pass (%)	37 (48.7)	62 (51.2)	0.771
Effective recanalization (%)	70 (92.1)	109 (90.1)	0.801
PTR time (minutes), mean (SD)	45.8 (24.5)	52.7 (31.0)	0.080
Overall SAH (%)	5 (6.6)	24 (19.8)	**0.012**
*With neurological deterioration* (%)	1 (1.3)	9 (7.4)	0.092
Good functional outcome (%)	25 (32.9)	31 (25.6)	0.330

ACAPCA, anterior cerebral or posterior cerebral artery; ASPECTS, Alberta Stroke Program Early CT Score; ICA, internal carotid artery; IQR, interquartile range; NIHSS, National Institutes of Health Stroke Scale; PTR, puncture-to-recanalization; SAH, subarachnoid hemorrhage; SD, standard deviation; SR, stent retriever; VABA, vertebral or basilar artery.

Table 2 presents a comparison of the frequency of post-procedural SAH, as well as symptomatic cases, across different occluded vessel locations between the Tron and conventional stent retriever groups. Notably, post-procedural SAH occurred more frequently in MCA occlusion (including both M1 and M2 segments) and MeVO cases. However, in MCA occlusions, both the incidence of overall SAH (including both asymptomatic and symptomatic cases) and SAH associated with neurological deterioration were significantly lower in the Tron group. Similarly, among MeVO cases (defined as occlusions involving the M2 segment, ACA, or PCA), the frequency of SAH was also significantly lower in the Tron group. Furthermore, when combining M1 occlusion and MeVO cases, both overall SAH (7.7% vs. 23.9%, *p* = 0.027) as well as SAH associated with neurological deterioration (1.9% vs. 12.7%, *p* = 0.044) were significantly reduced in the Tron group compared to the other stent retriever group.

**Table 2:** Comparison of the frequency of post-procedural SAH and those associated with neurological deterioration (symptomatic SAH), across different occluded vessel locations between the Tron and conventional stent retriever groups.

Occluded vessel	Overall SAH (%)			Symptomatic SAH (%)		
	Tron	Other SRs	*p*-value	Tron	Other SRs	*p*-value
ICA	6.3	13.5	0.655	0	0	0.99<
M1	11.1	22.5	0.232	2.7	10.0	0.99<
M2	0	25.9	**0.031**	0	14.8	0.99<
VABA	0	15.4	0.505	0	0	0.99<
ACAPCA	0	25.0	0.99<	0	25.0	0.99<
Overall	6.6	19.8	**0.012**	1.3	7.4	0.092
						
MCO^†^	7.8	23.9	**0.026**	2.0	11.9	0.076
MeVO^††^	0	25.8	**0.038**	0	16.1	0.150
MCO + MeVO	7.7	23.9	**0.027**	1.9	12.7	**0.044**

ACAPCA, anterior cerebral or posterior cerebral artery; ICA, internal carotid artery; MCA, middle cerebral artery; MCO, MCA occlusion; MeVO, medium vessel occlusion; SAH, subarachnoid hemorrhage; SR, stent retriever; VABA, vertebral or basilar artery.

^†^MCO includes occlusions in both the M1 and M2 segments.

^††^MeVO is defined as occlusions involving the M2 segment, ACA, or PCA. Bold values indicate statistical significance.

Table 3 presents the results of the multivariate logistic regression analysis for the incidence of post-procedural SAH. Use of Tron FX II remained independently associated with a reduced risk of SAH (adjusted OR 0.25; 95% CI 0.08–0.73; *p* = 0.006). In contrast, the number of stent retriever passes was independently associated with an increased likelihood of SAH (adjusted OR 1.72; 95% CI 1.12–2.63; *p* = 0.009). None of the other covariates demonstrated a statistically significant association with SAH; however, M1 occlusion showed a trend toward a higher risk of SAH compared with ICA occlusion (*p* = 0.075).

**Table 3:** Multivariate logistic regression analysis for predictors of post-procedural SAH (*n* = 197).

Variable	Adjusted OR	95% CI	*p*-value
Tron FX II use	0.25	0.08–0.73	**0.006**
Number of SR passes	1.72	1.12–2.63	**0.009**
SR size (per mm)	0.70	0.46–1.08	0.109
Age (per year)	1.04	0.99–1.11	0.148
Sex (female)	1.68	0.69–4.80	0.228
NIHSS (per point)	1.03	0.97–1.09	0.246
PTR (per min)	1.00	0.99–1.02	0.433
ASPECTS (per point)	0.94	0.75–1.18	0.624
Occluded vessel (ICA for reference)			
*M1*	4.01	0.90–17.74	0.075
*MeVO*	3.48	0.60–20.34	0.171
*VABA*	2.47	0.30–20.21	0.390

ASPECTS, Alberta Stroke Program Early CT Score; NIHSS, National Institutes of Health Stroke Scale; ICA, internal carotid artery; MCA, middle cerebral artery; MeVO, medium vessel occlusion; PTR, puncture-to-recanalization; SAH, subarachnoid hemorrhage; SR, stent retriever; VABA, vertebral or basilar artery. Bold values indicate statistical significance.

## 4 Discussion

Subarachnoid hemorrhage following MT has been associated with subsequent complications such as hydrocephalus and vasospasm, which may negatively impact clinical outcomes (4, 5). Although SAH is not always symptomatic, even asymptomatic cases may be associated with worse 90-day functional outcomes and higher mortality (28). Therefore, it remains a complication that should be actively prevented.

Our clinical results indicate that mechanical thrombectomy using a stent retriever with low radial and axial force (Tron FX II) significantly reduces post-procedural SAH in cases of MCA occlusion and MeVO, without significant differences in the used stent size or number of passes compared to conventional stent retrievers. Hemorrhagic complications following MT can be broadly classified into intracerebral parenchymal hematoma and extracerebral hematoma (29). While intracerebral hematoma is mainly attributed to reperfusion injury, extracerebral hematoma, represented by SAH, is largely associated with technical factors (30). Previous studies have suggested that such complications may result from vessel injury—particularly perforation or dissection—caused by vessel wall injury during device retrieval. This result of mechanical forces interaction is more likely to occur in distal, smaller, and tortuous vessels (4, 6, 9). Accordingly, MeVO may carry a higher risk of hemorrhagic complications compared to ICA, VA, or BA occlusions.

In stent retriever design, higher radial force increases contact pressure between the stent and the vessel wall, whereas greater axial stiffness increases the amount of longitudinal stress transmitted during retrieval—both of which may elevate the risk of endothelial injury. As a foundation for this clinical investigation, we first conducted a quantitative comparative analysis of the mechanical interaction parameters with the vessel wall for three widely used stent retrievers—Solitaire, Trevo, and EmboTrap—and compared them with Tron. Tron consistently demonstrated lower radial and axial forces applied to the vessel wall than the control devices. These bench test findings suggest that the Tron 4–40 may impose less mechanical stress on the vessel wall, particularly in tortuous vascular anatomy, and they provide the mechanistic rationale for the subsequent clinical study.

In our clinical study, 23.9% of patients treated with conventional stents experienced post-procedural SAH in M1 occlusion and MeVO, with half of these cases being symptomatic. This frequency of SAH is similar to that observed in the DISTAL trial (8). In contrast, the low radial and axial force stent retriever demonstrated a lower incidence of hemorrhagic complications in M1 and MeVO cases. Furthermore, use of Tron FX II was independently associated with a reduced risk of post-procedural SAH, as was a lower number of stent retriever passes. As our bench testing revealed that Tron exerts significantly lower radial pressure on the vessel wall, thereby minimizing vessel extension and displacement during retrieval and these mechanical properties likely contribute to a reduced risk of endothelial injury or vessel perforation, particularly in fragile or tortuous arteries. Our clinical findings support the suitability of the stent retriever with low radial force and enhanced axial flexibility for treating these cases, as they better accommodate vessel curvature and reduce mechanical strain on the vessel during retrieval.

Nonetheless, the low radial force stent retriever may provide weaker clot integration compared with higher radial force devices, particularly in cases involving firm or fibrin-rich clots. As a result, stand-alone use of Tron FX II may occasionally be less effective in removing particularly hard clots. This represents a trade-off for its advantage of reducing vessel wall stress and lowering the risk of procedure-related hemorrhagic complications. Accordingly, combined techniques such as CAPTIVE or SAVE may demonstrate greater efficacy when employed with Tron, given that clot entrapment in these methods primarily relies on the continuous negative pressure with the aspiration catheter rather than the radial force of the stent retriever (11, 12). In these techniques, the role of the stent retriever primarily serves to anchor the aspiration catheter, effectively retaining the thrombus between the catheter and the stent mesh, while also capturing the clot to prevent distal embolization. In this context, strong radial force may not be necessary. This is supported by our results, which showed no significant differences in the number of passes or effective recanalization rates between the low radial force stent and conventional stent retrievers.

Several limitations of this pilot study should be acknowledged. First, although the EVE model is engineered so that its friction, elasticity, and geometric properties closely approximate those of human arteries, it does not fully replicate all biological characteristics, which may introduce subtle differences from true *in vivo* conditions. Second, regarding clinical evaluation, the study was conducted at a single center by a single team. As with any consecutive cohort comparison, increased operator experience over time may have influenced the outcomes in the later Tron group. We also used various aspiration catheters for the combined technique, with selection based on vessel anatomy rather than stent retriever type. Although its impact on the incidence of SAH is likely minimal, variability in catheter selection may influence procedural performance and thus comparative outcomes. Third, multiple vessel-specific subgroup analyses were conducted without formal correction for multiple comparisons, consistent with the exploratory nature of this pilot study. In addition, the relatively small sample size, particularly in the MeVO subgroup, limits the statistical power to detect differences in secondary outcomes. Lastly, although Tron significantly reduced hemorrhagic complications, it did not lead to a significant improvement in long-term outcomes compared to conventional stent retrievers. This may be attributable to the small sample size and the older age of patients in our cohort, many of whom had impaired baseline activities of daily living, potentially limiting recovery even after technically successful reperfusion. Despite these limitations, our study provides the first clinical evidence that low radial and axial force stent retrievers can reduce the incidence of hemorrhagic complications in thrombectomy for MCA and MeVO occlusions. To further validate our findings, we plan to conduct a prospective, multicenter study to evaluate the clinical outcomes associated with the use of low radial and axial force stent retrievers.

## 5 Conclusion

The stent retriever with lower radial, axial, and retrieval traction forces was associated with a reduced incidence of post-procedural SAH and fewer adverse events in cases of MCA occlusion and MeVO. It also achieved effective recanalization rates comparable to those of conventional stent retrievers when combined with an aspiration catheter. These findings support the potential role of the low radial/axial force stent retriever as a safer option for treating occlusions in distal, small-caliber, and tortuous vessels, where minimizing vessel injury during retrieval is critical.

## Data Availability

The original contributions presented in the study are included in the article, further inquiries can be directed to the corresponding author/s.
